# Judging the Data: Peer Review versus Good Laboratory Practice Standards

**DOI:** 10.1289/ehp.120-a285a

**Published:** 2012-07-02

**Authors:** Charles W. Schmidt

**Affiliations:** Charles W. Schmidt, MS, an award-winning science writer from Portland, ME, has written for *Discover Magazine*, *Science*, and *Nature Medicine*.

When it comes to assessing the quality of toxicologic data to develop policy decisions, should regulators rely on journal peer review or on Good Laboratory Practice (GLP) standards? Authors of a review comparing the two conclude that the answer is neither. Instead, they propose that regulators need a well-defined scheme in which the best elements of both processes enable data to be weighed and evaluated in a consistent fashion [*EHP* 120(7):927–934; McCarty et al.].

The authors point out that peer review and GLP standards serve different but complementary needs. Traditional peer review aims to supply scientists with published articles worthy of consideration and debate; GLP standards—which are specific protocols for conducting and reporting experiments—aim to provide regulators with high-quality data that are acceptable across jurisdictions.

The authors go on to expose the shortcomings of each framework for policy use. According to their analysis, peer review suffers from reviewer bias, inconsistency in methods between journals, variable ability to identify fraud or falsified data, and a reluctance to publish results that show little or no effects. GLP standards, on the other hand, don’t address broad issues of scientific validity, although they supply consensual formats for gathering and analyzing data.

But instead of arguing over which of the two approaches best suits policy making, the authors propose that stakeholders should focus instead on the approaches’ growing convergence. That convergence, they propose, comes mainly from the peer-review side, which increasingly requires additional data reporting and supplementary methodological information for online publication—a trend they say serves public needs for data transparency and better communication of scientific concepts.

But what regulators in toxicology need above all else, the authors emphasize, is reliable, adequate, relevant data. Toward that end, the authors propose a weight-of-evidence scheme for data evaluation that comprises six steps: 1) Define the uses and goals of the regulatory action, and identify testable hypotheses; 2) define priorities for data weighting and support them with references; 3) gather the relevant data in a systematic way; 4) evaluate how well each selected study fulfilled its initial intent; 5) combine all data weightings in a predefined manner to achieve a score for each study; and 6) integrate the scores into a narrative that addresses judgments and conclusions derived from the entire evaluation process. Although neither peer review nor GLP standards can fully meet the needs for data quality and relevant science on their own, the authors write, a properly devised weight-of-evidence scheme can fill those overarching requirements.

